# Wearable Light Loggers in Field Conditions: Corneal Light Characteristics, User Compliance, and Acceptance

**DOI:** 10.3390/clockssleep6040042

**Published:** 2024-10-25

**Authors:** Oliver Stefani, Reto Marek, Jürg Schwarz, Sina Plate, Johannes Zauner, Björn Schrader

**Affiliations:** 1 Lucerne School of Engineering and Architecture, Lucerne University of Applied Sciences and Arts, 6048 Horw, Switzerland; reto.marek@hslu.ch (R.M.); sina.plate@gmx.ch (S.P.); bjoern.schrader@hslu.ch (B.S.); 2 Lucerne School of Business, Lucerne University of Applied Sciences and Arts, 6002 Lucerne, Switzerland; juerg.schwarz@hslu.ch; 3 Department Health and Sport Sciences, Chronobiology & Health, TUM School of Medicine and Health, Technical University of Munich, 80992 Munich, Germany; johannes.zauner@tum.de; 4 Translational Sensory & Circadian Neuroscience, Max Planck Institute for Biological Cybernetics, 72076 Tübingen, Germany

**Keywords:** light dosimetry, usability, daytime light exposure, evening light exposure, wearable light loggers, wearable technology, circadian lighting, corneal light exposure

## Abstract

Understanding user challenges with light dosimeters is crucial for designing more acceptable devices and advancing light exposure research. We systematically evaluated the usability and acceptability of a light dosimeter (lido) with 29 participants who wore the dosimeter near the corneal plane of the eye for 5 days. Common reasons for not wearing the dosimeter included exercise, recharging, wet environments, public places, and discomfort. Despite these issues, participants adhered to using the dosimeter with high compliance (89% of recording time). Our findings revealed a significant discrepancy between mean (300 lx_mEDI_) and median (51 lx_mEDI_) melanopic equivalent daylight illuminance. This discrepancy indicates that the participants were exposed to significantly lower light levels most of the time. Specifically, participants were exposed to light levels above 250 lx_mEDI_ for only 14% of their wearing time. This highlights the need for increased exposure to recommended light levels. In the evening, participants were exposed to less than the recommended 10 lx_mEDI_ for 58% of their wearing time, which is in line with the guidelines for reducing light exposure before sleep. This study highlights the urgent need for strategies to increase daily light exposure that are more in line with circadian health recommendations.

## 1. Introduction

The evolution of circadian clocks and adaptation to the 24 h light/dark cycle occurred under natural conditions long before the invention of electricity and the use of light sources other than the sun. Circadian clocks are internal biological mechanisms that regulate the circadian rhythm of various physiological processes in living organisms, including humans. In fact, indoor work environments typically have significantly different lighting characteristics than outdoor environments [1]. These differences co-determine the personal light exposure of occupants, which in turn can affect their health and well-being. These differences include the temporal and spatial dynamics, spectrum, and intensity of light. Outdoors, people are exposed to strong contrasts in illuminance. Compared to static lighting indoors (static ~500 lx [1,2]), a sunny day at noon can be a hundred million times brighter than a moonless night. Numerous studies have verified the effects of light on physiology (e.g., sleep–wake timing, hormone release [3,4,5,6], and metabolic processes [7,8]). Additionally, light influences behavioral functions such as alertness, mood, and cognitive performance [9,10,11,12,13,14,15]. How the results of laboratory studies translate to the real world, however, is not yet fully understood since there is a lack of field studies investigating light’s effects on humans [16]. Open questions include if can we collect enough light with a bright “daylight shower” within a few minutes, or if we should collect lower but sufficient continuous light levels throughout the day to support a healthy lifestyle. Is it the time above certain thresholds, the total light dose (the area under the curve (AUC)), the median, the mode, or the mean illuminance that matters? Although the full implications are still being explored, tracking light exposure over extended periods using wearable light loggers is crucial for gaining real-world insights and linking these patterns to physiological outcomes. Early evidence from field studies has shown that the availability of natural light can significantly impact sleep quality and sleepiness: office workers with access to windows report better sleep quality than those without [17]. Sleep quality tends to improve as daylight availability increases, particularly in summer when days are longer and brighter [18]. More light during the day, especially for durations above certain thresholds such as 1000 lx or 2500 lx at eye level, was associated with better sleep quality [19]. In addition, previous exposure to light for several hours was associated with less subjective sleepiness, with brighter light during the late sleep phase and after waking being associated with less early morning sleepiness [20]. To be able to give better advice on lighting behavior, such as whether it is better to spend short periods outdoors in bright light or to spend the whole day in moderately high illuminance, we need to back up these findings with more evidence. Metrics recorded with wearable light loggers should include the melanopic equivalent daylight illuminance (mEDI) [21] along with auxiliary data to identify periods of nonwear (e.g., tilt angle of the device) [22].

The acceptance of light loggers is essential to ensure their correct and consistent use. If wearers find them uncomfortable, socially awkward to wear, or cumbersome, the likelihood of noncompliance increases, which in turn compromises data quality. While wrist-worn devices are less obtrusive, they have limited reliability for accurately assessing the light dose at the eye, which is critical for understanding the non-image-forming (NIF) effects of light. This discrepancy occurs because wrist-worn sensors capture light from a different plane [23,24,25]. The accuracy of wrist-worn devices is not only affected by the different measurement positions but also by potential obstruction by clothing, leading to significant deviations from the actual light exposure at the eye, partly dependent on climate and weather. This limitation is particularly important in the evening when small variations in ambient lighting can profoundly affect NIF effects. Therefore, to obtain the most relevant and reliable data, these devices must measure the light received as close as possible to the eye [22,26,27].

While the accuracy of a measuring device is very relevant, acceptance as a nonfunctional requirement is also important. Regardless of its accuracy, if a device is not fully accepted by a participant or their environment, it may not be used consistently. A participant’s wear compliance, however, is also influenced by factors such as how the device was introduced to them and their motivation to participate in a study (e.g., intrinsic motivation or compensation). Light dosimeters attached to spectacle frames are highly visible, which can cause discomfort or self-consciousness in participants [28]. This factor might be particularly important for people who do not normally wear glasses, as they may find devices attached to glasses even more cumbersome, leading to lower compliance rates. In addition, prolonged use in multiday studies that require continuous wear of light dosimeters may result in higher dropout rates or noncompliance, compromising the integrity of the data collected. Understanding the challenges users face is essential for designing more universally acceptable devices. Researchers can design studies with better compliance strategies and improve data quality by understanding the likelihood of noncompliance and the specific challenges participants face. In this project, we systematically evaluated the factors influencing the usability and acceptability of a light dosimeter “lido” [26], focusing on its practical use in field studies. These factors provide evidence-based advice to researchers. In addition, the findings will inform future improvements in the design of light dosimeters to increase their convenience and usability for diverse participant groups. Although the cleaning of recorded light exposure data is a critical step when assessing personal light exposure, there is a notable lack of systematic studies investigating appropriate cleaning methods [22]. In our study, we implemented four steps to clean the recorded data. In addition to exploring the acceptability of device wear, our study aimed to analyze the summarized results of the actual device measurements and interpret their potential significance.

## 2. Results

### 2.1. Reasons for Not Wearing the Dosimeter

The reasons for not wearing a lido varied widely and were categorized using thematic analysis [29]. The full list of reasons is provided in Supplement S1. Common reasons included, for example, attending public events such as business dinners, conferences, presentations, going out, and meetings (summarized as “public” below). The reasons for not wearing outside of sleeping and showering were

- Charging battery (5×);
- Wetness in the environment (9×);
- Public (14×);
- Pain from lido (9×);
- Sport (11×).

Most participants (*n* = 16) reported not wearing the device for a few hours. Possible answers for nonwear time in the form were a few minutes, a few hours, and several hours. The objectively assessed wear time for these participants was 49% of the total recording time (total recording time in this case included the recording time during sleep). Ten participants reported removing the lido for only a few minutes. The objectively assessed data showed that these participants did wear the lido for 59% of the total recorded time. Only three participants reported removing the lido for several hours. For these participants, the objective data showed that they did wear the lido for 40% of the total recorded time.

Notably, none of the participants completely forgot to wear the light dosimeter. Participants provided specific reasons in their diaries when the devices were not worn. There was only one case where a participant forgot to document the period of nonwearing. Participants were diligent about wearing the dosimeter and pressing the trigger button when they removed the device. This was verified through analysis of the tilt data, light measurements (e.g., the mEDI (melanopic equivalent daylight illuminance)), and the trigger events.

### 2.2. Feedback About the Usability of the Devices

Participants often noted that they received skeptical looks from bystanders in public, presumably because the device’s appearance resembled that of a camera, as participants reported. This led to privacy concerns and unwanted attention. On the positive side, some participants noted that the device sparked interest in this study, with some people expressing a desire to participate.

Many participants mentioned that the device (with a weight of 27 g) caused an imbalance when attached to their glasses, particularly when walking. This imbalance affected their comfort and ease of movement. Other aspects of wearing the device were generally well tolerated, although continuous wear over several days was challenging for some due to the lack of overall comfort (see Supplement S1 for details). Key issues identified through the thematic analysis included

- Many looks from others (18×);
- Interest of others (15×);
- Looks like a camera (14×);
- Imbalance (5×);
- Positive reactions from others (3×);
- Does not look good (2×);
- Glasses pinching (2×);
- You become accustomed to wearing it (1×);
- LED flashes too bright in the dark (1×);
- No reaction from strangers (1×);
- Awareness of personal light exposure (1×).

The results of this study can be compared to those of Kelly and Gilbert (2016) [30], although it is important to note the different Likert-type scales: Kelly and Gilbert (2018) [31] used a six-point scale, while this study used a five-point scale with a neutral midpoint of 3 = “neither”. As a result, a direct comparison of the WEAR Scores is only possible with certain limitations. The comparable, linearly transformed WEAR Score from this study to the six-point scale is 3.50 versus 3.55 in Kelly and Gilbert (2018) [31] (see Table 2).

For comparison with Balajadia et al. (2023) [28], the WEAR Score of this study was also linearly transformed into the seven-point scale and resulted in 4.60, which is significantly higher than the WEAR Score of 4.00 in Balajadia et al. (2023) [28].

### 2.3. Recording Times and Wear Compliance

**Figure 1:**
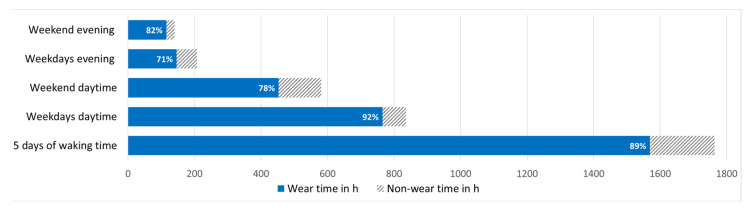
Wearing times (blue bars) as a function of day of week and time of day. Hatched grey bars represent nonwear times. Total length represents recording time excluding sleep. Excluding sleep and the habitual 3 h before going to bed, compliance was high, at 92% between Wednesday and Friday (weekdays) during daytime. On weekends in the evening, lidos were worn 82% of the recording time. The total length of the “5 days of waking time” bar depicts the total recording time excluding sleep.

**Figure 2:**
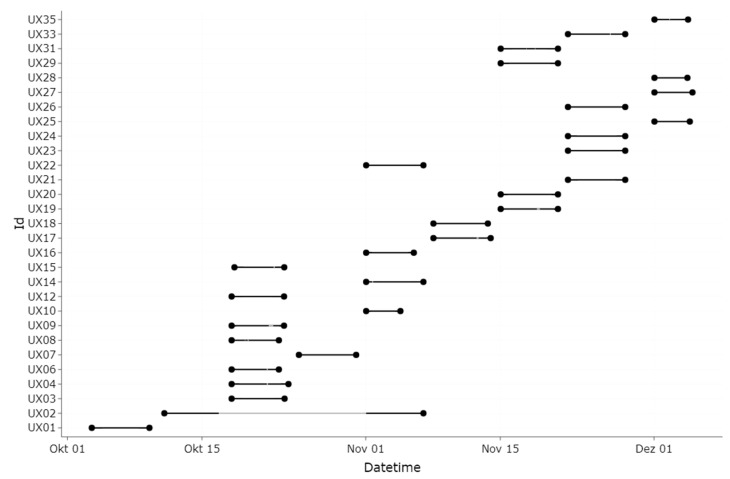
Timeline (x axis) of the experiment and when a lido was worn by a participant. A total of 29 participants took part in the experiment (y axis). Participant UX02 stopped wearing the lido due to illness. As the same lido was assigned to UX02, the grey line indicates nonwear time. The lido was worn for five consecutive days from 1 November onwards. UX10 stopped wearing the lido during the fourth day because the device was out of battery and could not be charged anymore. UX07 became sick during the first scheduled wearing time and stopped wearing the device.

Due to technical issues, we recorded 3031 h out of a potential 3480 h (29 participants × 24 h × 5 days). During that recorded time, lidos were worn for 1569 h, representing 52% of the total recording time (total recording time included ~8 h recording during night-time when the lido was placed next to the bed). During waking hours, lidos were worn 89% of the recording time. Figure 1 depicts an overview of the recording times and the wear compliance. The total wear time in the evening on weekdays was 164 h of the 207 h recording time (79%). The total recording time during the evening on weekends was 140 h, of which the lidos were worn 115 h (82%). On weekdays, lidos were worn for 92% of the daytime recording time (836 h) from waking up to 3 h before bedtime (the 3 h before bedtime are referred to below as the evening). The total recording time during the week (Wednesday to Friday), including evenings and bedtime, was 1777 h. On weekends, lidos were worn for 78% of the daytime recording time (453 h). The total recording time during the weekend was 1255 h. Participants wearing glasses (*n* = 20) wore the lido for 52% of their total recording time, including evening and bedtime (1090 h). Participants not normally wearing glasses (*n* = 9) wore the lido for 51% of their total recording time (479 h). Sex differences were minimal, with women wearing a lido 52% and men 51% of the total recording time. None of the moderate morning types (as assessed by the MEQ) wore the lido after midnight, and none of the moderate evening types wore the lido before 8:30 am. Participant UX7 only wore the device for two days because of illness (Figure 2). Toward the end of the experimental phase, participants UX25, 27, 28, and 35 wore the lido for five consecutive days (according to their diary). For unknown reasons, the lidos only recorded from Friday onward, although they were programmed to record from Wednesday onward.

### 2.4. Light Characteristics Received by Subjects near the Plane of the Cornea

**Figure 3:**
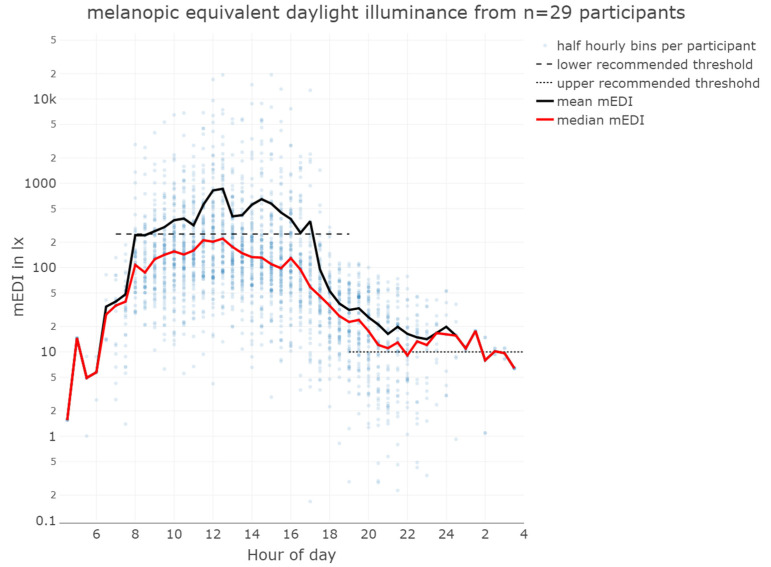
Mean and median melanopic equivalent daylight illuminance from 29 participants wearing the lido for five consecutive days. There was a significant difference between the mean values (black line) and median values (red line). The dashed line indicates the lower limit of recommended daytime light exposure, and the dotted line indicates the upper limit of recommended evening light exposure.

The overall median mEDI at the eye level while wearing the lido was 51 lx_mEDI_, with a mean of 299 lx_mEDI_, minimum 0 lx_mEDI_, first quartile 11 lx_mEDI_, third quartile 138 lx_mEDI_, and maximum 93,432 lx_mEDI_, indicating variability in light exposure among the participants (see Table 1 and Figure 3). The very high illuminances, approaching 100,000 lx_mEDI_, resulted in elevated mean illuminances that may not accurately represent the received light dose. In contrast, median illuminances, or the duration of illuminance above a certain threshold, may give a more accurate picture of cumulative light exposure history.

**Figure 4:**
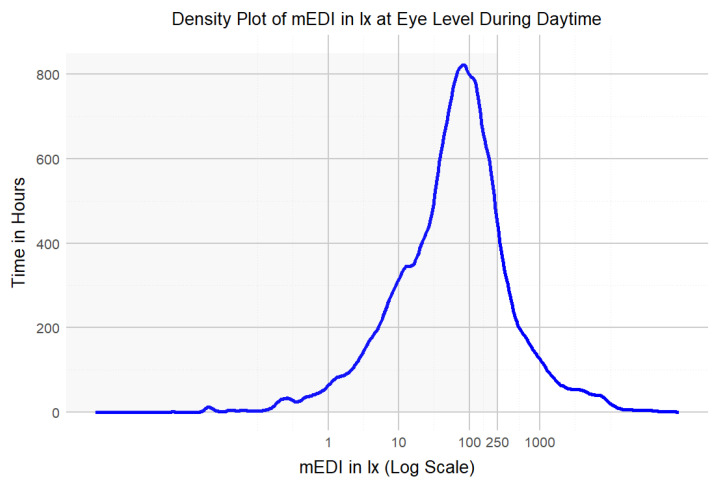
Density plot of mEDI in lx and time in hours during daytime. The grey area depicts light levels below the recommended 250 lx_mEDI_. The time in hours was calculated by multiplying the number of measurements by 360, as the measurement interval was every 10 s.

**Figure 5:**
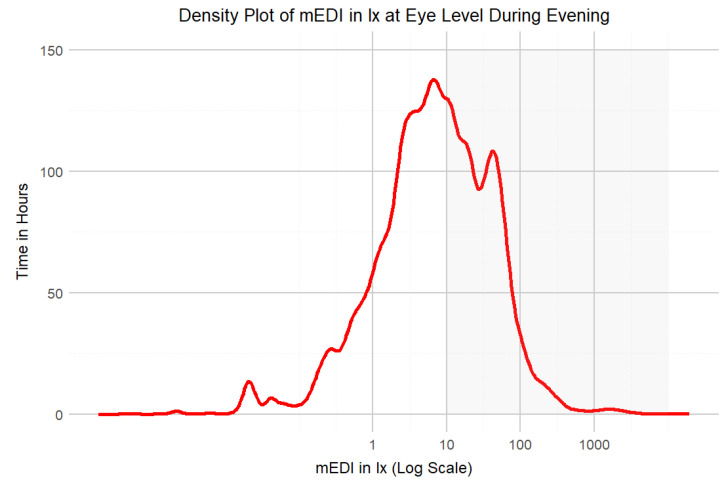
Density plot of mEDI in lx and time in hours during the evening (i.e., 3 h before bedtime). The grey area depicts light levels above the recommended 10 lx_mEDI_. The time in hours was calculated by multiplying the number of measurements by 360, as the measurement interval was every 10 s.

**Figure 6:**
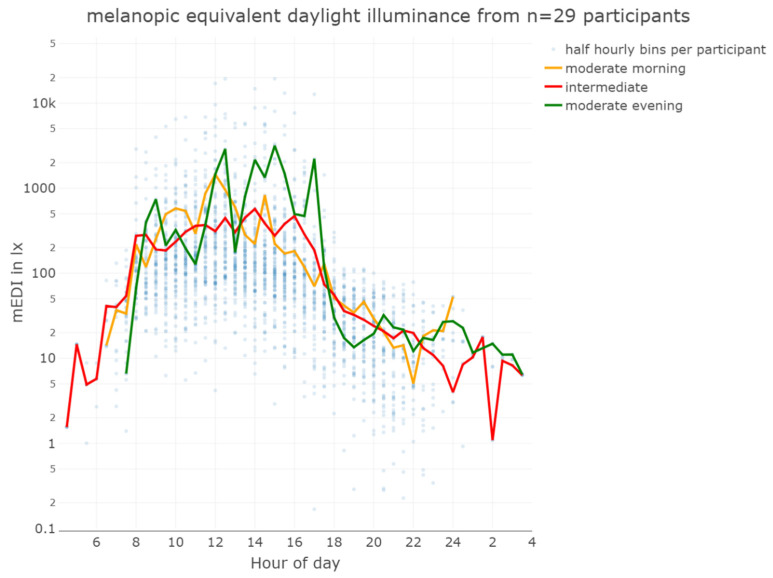
Mean equivalent daylight illuminance from 29 participants wearing a lido for five consecutive days. Moderate evening types recorded no light exposure at the eye level before 7:30 AM, and moderate morning types did not record light exposure after midnight.

During daytime (excluding the evening), the participants achieved light exposure levels above 250 lx_mEDI_ [32] for only 14% (224 h) of the total wearing time, with similar percentages on weekdays (15%) and weekends (13%). This highlights the need for increased exposure to recommended light levels. Time above 250 lx_mEDI_ during wearing time (excluding sleep but including the evening) was 18% (132 h) on weekdays and 17% (75 h) on weekends. Most of the time, the participants were in light levels below 100 lx_mEDI_ (Figure 4). In the evening, the participants’ light exposure was below the recommended 10 lx_mEDI_ for 57% (93 h) of the wear time on weekdays and 58% on weekends, in line with the guidelines for reducing light exposure before sleep [32] (Figure 5, Table 1). Unobstructed outdoor illuminance measurements revealed light levels exceeding the recommended daytime values already in the early morning, with peaks over 50,000 lx at mid-day. Although the participants may not have spent all their time outdoors and the outdoor light measurements cannot be directly correlated with individual participants, the outdoor illuminance in Switzerland, even during autumn, consistently exceeds the recommended 250 lx_mEDI_. Figure 6 depicts the light conditions (mEDI) for the three different chronotypes in our study. The trend suggests higher light exposure for moderate evening types in the afternoon than for moderate morning types. Still, the statistical analysis revealed no significant differences between the daily light patterns of the chronotypes (see Supplement S2 for details). The participants showed a significant difference in wake-up times (pick-up time of lido) between weekdays and weekends, with an average delay of 1 h and 36 min on weekends. The average first-wear time of the lidos after wake-up was 07:40 AM during the week and 09:16 AM. on the weekend. There was a ca. 40 min standard deviation between participants and a standard deviation of ca. 41 min between weekends and weekdays.

## 3. Discussion

After excluding the time spent sleeping, during which participants were not required to wear the device, compliance was notably high, with 89% wear time during waking hours. This high level of compliance was crucial for ensuring the quality and reliability of the data collected. No participant forgot to wear the device completely, and all were careful to document periods of nonwear and to press the trigger button when removing the device. Nevertheless, when wearing the lido, participants often noticed skeptical looks from those passing by, which raised privacy concerns and caused unwanted attention. Furthermore, the device caused imbalance and sometimes even pain when attached to spectacles. Therefore, continuous wear over several days posed a challenge to some participants.

Despite participants reporting unwanted attention in public and significant discomfort while wearing the device, compliance rates during daytime hours were high, with participants wearing the lido for 92% of the time on weekdays and 78% on weekends. This suggested robust overall adherence to the wear protocol. We attribute this high level of compliance not to the extrinsic motivation of avoiding a 40% reimbursement penalty for removing the dosimeter for more than six hours per day but rather to the intrinsic motivation of contributing to scientific research, as emphasized during the briefing. It is worth noting that only one participant did not receive the full reimbursement as he could not wear the device due to illness.

The difference in wear time between weekends and weekdays suggests that participants’ routines and weekend activities lead to lower compliance, which could be addressed by targeted strategies to maintain consistent wear time. There were negligible differences in compliance between participants who wore glasses and those who did not. Sex-based differences were also small, with women wearing the device 52% of the time compared to 51% for men.

The WEAR Score, which measures the social acceptability of wearable devices, is comparable to the score reported by Kelly and Gilbert [31]. However, it is significantly higher than the score in the Balajadia et al. [28] study, indicating greater social acceptability in the current context. Several factors could contribute to the higher score, such as the greater technological affinity of students from a technical university (the majority of our cohort) compared to those from Oxford University, the motivational incentive of informing participants that the device was developed at their own university, and the extended period (5 days) that participants wore the device in our study, which allowed them to become accustomed to it. In addition, Balajadia et al.’s use of a reduced set of 41 items compared to the original 50 items in our study may have introduced more of a negative bias into their results. Our study cohort consisted entirely of young people, which may have introduced bias into the results. This age group may either be more concerned with their appearance or more open to adopting innovative devices than an older population. In addition, as Kelly and Gilbert [31] noted, individuals who need a wearable device to support their health may have higher levels of acceptance. In their study, a head-worn device received a lower WEAR Score of 3.25 when categorized as a fitness device, compared to a higher score of 3.76 when categorized as a medical device [31]. As a result, our findings may not fully represent the attitudes and behaviors of other populations.

The significant variation in wake-up times between weekdays and weekends (ca. 1.5 h later on weekends) corroborates previous findings by Wittmann at al., namely, “social jetlag” [33]. Social jetlag refers to the misalignment between an individual’s internal biological clock and their socially imposed schedules, such as work or other commitments. It occurs when a person’s natural sleep–wake cycle, dictated by their internal body clock, does not align with the timing demands of their daily activities.

The analysis of the light exposure data from this study showed that there was considerable variability in the light exposure of the participants. While the (arithmetic) mean mEDI of almost 300 lx_mEDI_ suggests that participants generally received adequate light during the day, the median mEDI of only 51 lx_mEDI_ shows that, most of the time, light exposure was much lower. This discrepancy was due to occasional very high maximum illuminances of almost 100,000 lx_mEDI_, which skewed the (arithmetic) mean. The overall geometric mean with 50 lx_mEDI_, however, was less sensitive to extreme outliers, too. The geometric means in the evening and during daytime were 6 and 57 lx_mEDI_, respectively.

Given the potential importance of continuous light exposure at recommended thresholds, it is crucial to examine the duration of time participants spent above certain light thresholds. The recent recommendations [32] suggest a minimum of 250 lx_mEDI_ during the day. Previous studies investigating photopic illuminance at the wrist, however, reported that participants of different ages experienced illuminance levels below 100 lx for more than 50% of their waking hours [34]. In our study, most of the day was spent below 100 lx_mEDI_, indicating insufficient exposure to the light levels recommended in the literature. Specifically, the participants were exposed to light levels above 250 lx_mEDI_ for only 14% of their wearing time, with a slight increase on weekdays (15%) compared to weekends (13%). This suggests a need for strategies to increase light exposure. The data were collected during the autumn and winter months, which likely contributed to the low levels of light exposure observed. Light exposure is likely higher in summer, as other studies have reported increased light exposure during this season [35,36,37].

Looking at wear time excluding sleep but including evening periods, the exposure to light above 250 lx_mEDI_ was slightly higher: 18% on weekdays and 17% on weekends. While this may seem like an improvement, it is problematic because the increase in light exposure above this threshold occurred three hours before bedtime, a time when light levels should ideally be lower than 10 lx_mEDI_. However, in the evening, the participants were generally more likely to comply with the recommendations to reduce light exposure before going to bed. The participants’ light exposure three hours before bedtime was below the recommended 10 lx_mEDI_ for 57% of the wearing time on weekdays and 58% on weekends, in line with the guidelines aimed at minimizing light exposure before bedtime to support a healthier lifestyle [32]. These results may not be generalizable to the broader population due to the potential biases in our participant sampling, which could affect the representativeness of light exposure patterns.

## 4. Materials and Methods

### 4.1. Participants and Procedure

The study procedures were approved by the local ethics committee and conducted in accordance with the Declaration of Helsinki. Written informed consent was obtained from all participants. Participants were recruited through a combination of a physical flyer displayed at our university and a LinkedIn post with the same information. Both methods provided details about this study and invited interested individuals to participate. We recruited 29 participants (17 women) aged between 18 and 35 years to wear lido devices for five consecutive days between 8 October and 4 December 2023. Of these participants, 20 were spectacle wearers. Participants could follow their daily routines and were instructed to wear the device as much as possible to accurately monitor their light exposure for scientific purposes. To further ensure compliance, the financial compensation was reduced by 40% if participants did not wear the device for more than six hours during the day (excluding night-time when participants placed the lido next to their bed). Each participant began wearing the device on a Wednesday morning directly after waking up. After five days of wearing the lido, participants received an email on Sunday evening with a link to answer questions about their experience with wearing the device.

### 4.2. Instruments

The online questionnaire was created in Qualtrics and included standardized questionnaires such as the MEQ (Morning Evening Questionnaire) [38] and the WEAR (Wearable Acceptability Range) Scale [31]. The WEAR Scale is an instrument used in consumer behavior research and product development to assess the social acceptability of wearable devices [31,39]. The original form of the WEAR Scale consists of 50 items, each rated on a 6-point Likert-type scale ranging from 1 = “strongly disagree” to 6 = “strongly agree” [31,39]. In her dissertation, Kelly (2016) [39] demonstrated that the 50 items of the original WEAR Scale could be condensed into a construct known as the WEAR Score, characterized by two factors: (1) “fulfillment of aspirational desires” (8 items) and (2) “absence of social anxiety” (6 items). The WEAR Score ranges as well from 1 = “strongly disagree” to 6 = “strongly agree”, with a mean of 3.5.

Likert-type scale scores are adjusted based on context and specific questions. For example, Balajadia et al. (2023) [28] used a 7-point WEAR Scale in their study, ranging from 1 = “strongly disagree” to 7 = “strongly agree”. This modification introduced a neutral midpoint, 4 = “neither agree nor disagree”. The present study used a 5-point WEAR Scale, ranging from 1 = “agree” to 5 = ”disagree”, with a neutral midpoint of 3 = neither, to maintain consistency with the other five-point Likert-type scales used in our research. Table 2 gives an overview of the different Likert scales used in the mentioned studies.

We further collected feedback from participants about their experiences with the light dosimeters, focusing on what worked well, what did not, and what could be improved. This feedback was analyzed thematically, following the steps proposed by Braun and Clarke [29] to identify common themes and potential areas for improvement. To address our particular interest in the usability and acceptability of the lidos, we included the following open questions:

- Please tell me when and where you mainly wore the lido.
- What activities did you do while you were carrying it?
- Aside from times when you slept and showered, how long did you not wear the lido when you could otherwise have worn it (time of the day)?
- What were the possible reasons for not wearing the lido, apart from the times when sleeping and showering?
- On which occasion did you find it particularly difficult to wear the lido?
- What experiences (your own experience and possibly also the reactions of other people) have you had while wearing the device? Please briefly describe your observations.
- When and where would you prefer to wear the lido?

### 4.3. Apparatus

**Figure 7:**
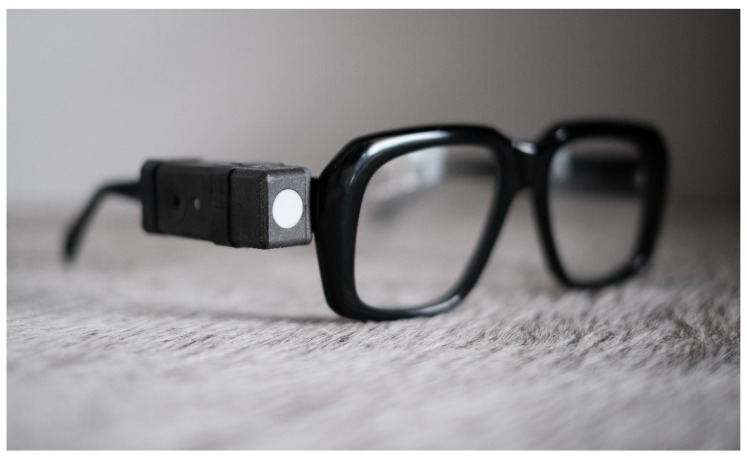
The wearable light dosimeter, “lido”, attached to a pair of glasses. The white circle is the diffusor. The small grey circle on the left is an indicator light that flashes green every 10 s (0.1 Hz) when recording.

A light dosimeter (lido) [26] is a wearable instrument designed to measure light exposure, as shown in Figure 7. When attached to a pair of eyeglasses, it captures time-series data (every 10 s) of light exposure in the near-corneal plane using the metrics defined in CIE S 026:2018 [21]. The acquired data, e.g., the five α-opic EDIs (equivalent daylight illuminances), can be processed directly in LidoStudio (proprietary windows application specifically developed for the lido device) or exported as a comma-separated values (CSV) file for further analysis. The light dosimeters have been rigorously tested and validated by the Swiss Federal Institute of Metrology (METAS).

### 4.4. Data Preprocessing

The data preprocessing to determine nonwear and sleep times was conducted in four steps, driven by a specific interest in quantifying the actual amount of light reaching participants’ eyes near the corneal plane:

*1. Automated Tilt Angle Analysis for Nonwear Detection:* An automated method using R [40] was utilized to infer nonwear periods based on the stability of the device’s tilt angle. This approach involves computing rolling averages of tilt angles over one-minute intervals, encompassing six measurements per interval. These rolling means are then compared with the actual tilt angles recorded. If the absolute difference between the rolling mean and the actual tilt angle remains under 3 degrees for a continuous period of at least three minutes, this stability is interpreted as an indication that the device was not worn, leading to the exclusion of these light measurements from further analysis.

*2. Visual Inspection for Light Level Anomalies:* The accuracy of wear-time analysis is enhanced through a visual inspection of light measurements. This process specifically targets and removes data where light levels are recorded at less than 1 lx during daylight hours. This step is crucial, as algorithmic analysis may not identify instances where the device was stored in locations like a pocket or bag, which could mimic a wear situation.

*3. Verification of Nonwear Times Through Trigger Events and User Logs:* Periods identified as nonwear were corroborated through a manual verification process that utilized trigger times logged and detailed entries in user diaries. This involved a careful examination of the reported times when the device was explicitly set aside, adding an additional layer of validation to the automated and visual inspection methods. All reported nonwear time ranges were removed manually if the automatic algorithms mentioned above had not yet removed them.

*4. Estimation of Sleep Times Based on Last Known Movement:* Sleep periods were estimated by analyzing the timestamps of the last recorded movement each day, coupled with entries from user diaries. If no movement was detected post 8 PM and the device was not picked up before 7 AM the following morning, these intervals were categorized as sleep times. Records indicating that the device was set down before 8 PM without subsequent user interaction or diary notation to the contrary were considered implausible for sleep time and marked as ‘not applicable’ in the dataset.

**Figure 8:**
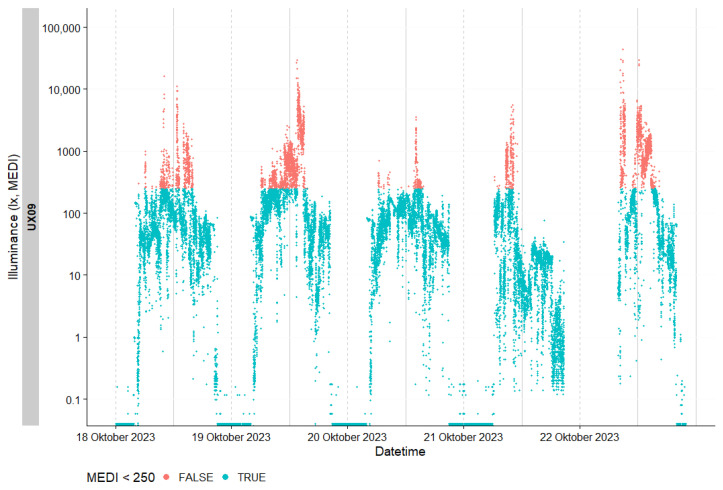
Individual light exposure of one exemplary participant before cleaning data according to nonwear times. Depicted in orange are the light levels above the recommended 250 lx_mEDI_.

**Figure 9:**
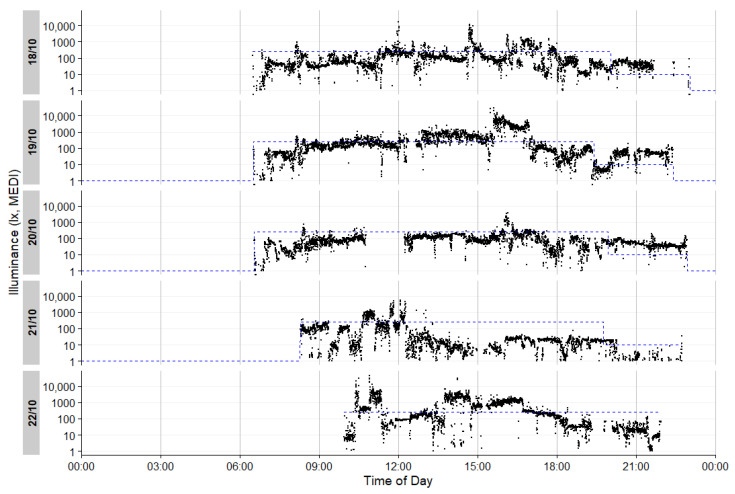
Individual light exposure of one exemplary participant after cleaning data according to nonwear times. The y axis depicts mEDI in lx for each of the 5 days. The x axis depicts the time of day. The blue dotted line indicates the 250 lx_mEDI_ level during the day and the 10 lx_mEDI_ level 3 h before bedtime (adjusted for individual bedtime).

The last three hours before each participant’s habitual bedtime were calculated individually and defined as evening. Daytime was determined individually from waking up until three hours before bedtime. Generally, the “melanopic equivalent daylight illuminance” (mEDI) [21,41] is reported as this measure has been shown to be the best predictor of the non-image-forming (NIF) effects of light [42,43,44]. Figure 8 presents the raw individual light exposure data for a representative participant prior to the exclusion of nonwear periods. Light levels exceeding the recommended threshold of 250 lx_mEDI_ are highlighted in orange. Figure 9 illustrates the individual light exposure for the same participant after data cleaning to account for nonwear periods. The analysis and visualization were performed using LightLogR [45] software, version 0.4.2.

### 4.5. Statistics

Data were analyzed and visualized with R statistical software [40] (Version 4.4.0) and the LightLogR [45] package (version 0.4.0). Inferential analysis was performed with generalized additive mixed models (GLMMs) [46,47] through the mgcv [47] (Version 1.9.1) package. Specifically, a cyclic (24 h) smooth was fitted to the logarithmic (base 10)-transformed values of melanopic EDI, with participants as random effects. To test the effect of chronotype on the daily light patterns, the five theoretical model types according to Pederson et al. [48] were implemented and compared through Akaike’s information criterion (AIC), as suggested by several sources on GAM(M)s [47,48,49]. Following the example of Pedersen et al., models that differed by two units or less from the lower AIC had substantial support with the more parsimonious model to be preferred.

## 5. Conclusions and Recommendations

We conducted a thematic analysis of participants’ reasons for not wearing a light dosimeter (lido). The primary reasons identified included attending public events, needing to recharge the battery, exposure to wet environments, discomfort with the device, and engaging in exercise. Despite these challenges, compliance remained high, with 89% of wear time during waking hours.

Recognizing that compliance is influenced by various factors beyond the device itself, user challenges can be mitigated through clear instructions, support, and appropriate user guidance. Effective compliance strategies should include thorough training to educate users about the importance of consistent device use, complemented by motivational incentives, both intrinsic (e.g., contributing to scientific research) and extrinsic (e.g., financial incentives, personalized reports).

To accurately identify nonwear periods, continuous monitoring and support systems should be implemented, such as auxiliary data from actimetry or tilt sensors. These systems can promptly address problems, reinforce proper usage habits, and assist in cleaning data for nonwear periods. Additional biometric measurements, such as skin conductance, temperature, or heart rate, could further detect nonwear times and improve data accuracy. While participant diaries help with contextualizing the data collected, the integration of automated supplementary data can reduce the burden on participants.

An unobtrusive and innovative design could make eye-worn devices less noticeable to both the wearer and others in public settings. Future designs should aim to minimize size and weight while improving aesthetics and avoiding a camera-like appearance to increase acceptance and compliance. Exploring alternatives to spectacle frame attachments could also make devices less obtrusive when worn near the eye. Addressing the specific challenges faced by different demographic groups, such as aesthetic concerns in young adults, can improve device usability, participant comfort, and compliance. Additionally, developing adaptable fitting methods and incorporating waterproofing features for those who engage in outdoor exercise could further enhance usability and acceptance.

Although the mean melanopic equivalent daylight illuminance was nearly 300 lx_mEDI_, suggesting adequate light exposure during the day, the median mEDI was only 51 lx_mEDI_. This discrepancy indicates that, for most of the time, the participants experienced significantly lower light levels. Specifically, the participants were exposed to light levels above 250 lx_mEDI_ for only 14% of their wearing time. Wearable light dosimeters offer a practical solution for collecting granular data on individual light exposure in everyday environments, revealing patterns that cannot be seen in laboratory-based experiments. Better dosimeters could improve public health strategies and ensure that more people receive the light they need for better health.

## Figures and Tables

**Table 1:** Light characteristics at eye level of 29 participants during 5 days in autumn in Switzerland.

mEDI in lx	Mean	Median	Minimum	1st Quartile	3rd Quartile	Maximum
daytime	356	67	0	20	165	93,432
evening	32	7	0	2	22	19,376
overall	299	51	0	11	138	93,432

**Table 2:** Overview of different Likert scales for the WEAR Score used in various studies.

Options	5-Point Likert Scale	6-Point Likert Scale	7-Point Likert Scale
	(current study)	(Kelly [31,39])	(Balajadia et al. [28])
Strongly disagree	1	1	1
Disagree	2	2	2
Somewhat disagree		3	3
Neither agree nor disagree	3		4
Somewhat agree		4	5
Agree	4	5	6
Strongly agree	5	6	7

## Data Availability

The raw light measurement data, along with the cleaned dataset used for analysis, will be made available on Zenodo upon acceptance of this manuscript. A DOI for the Zenodo repository will be provided.

## Supplementary Materials

The following supporting information can be downloaded at: https://www.mdpi.com/article/10.3390/clockssleep6040042/s1, Supplement S1: complete participant responses: reasons for not wearing and additional factors related to wearing lidos per participant; Supplement S2: analysis of differences in light exposure patterns between chronotypes.
