# Supplementary material for: Wearable Light Loggers in Field Conditions: Corneal Light Characteristics, User Compliance, and Acceptance
Source: Clocks Sleep. 2024 Oct 25;6(4):619–34. doi: 10.3390/clockssleep6040042 (PMC11586969; doi:10.3390/clockssleep6040042)
Supplement: Supplementary file 1 [file clockssleep-06-00042-s001.zip › supplementS1.pdf]

Wearable light loggers in field conditions: Corneal light characteristics, user compliance and acceptance

Oliver Stefani [1*], Reto Marek [1], Jürg Schwarz [2], Sina Plate [1], Johannes Zauner [3,4] and Björn Schrader [1]

[1] Lucerne School of Engineering and Architecture, Lucerne University of Applied Sciences and Arts, Horw, Switzerland; oliver.stefani@hslu.ch
[2] Lucerne School of Business, Lucerne University of Applied Sciences and Arts, Horw, Switzerland; juerg.schwarz@hslu.ch
[3] Technical University of Munich, TUM School of Medicine and Health, Department Health and Sport Sciences, Germany; johannes.zauner@tum.de
[4] Max Planck Institute for Biological Cybernetics, Max Planck Research Group Translational Sensory & Circadian Neuroscience, Tübingen, Germany
* Correspondence: oliver.stefani@hslu.ch

Supplement S1

Complete participant responses: Reasons for not wearing and additional factors related to wearing lido per participant (translated from German).

Please tell us in bullet points when and where you mainly wore the LiDo:

1. at school during the day, in your free time from Friday, while doing homework
2. at home, at university, at work, while driving, while working on your laptop
3. at school, while studying in front of the PC
4. all day long. At the university. At home. Free time
5. all day, only when showering, at home (Fr home office, otherwise free time), walking, shopping, in the restaurant, driving
6. Mainly at school, at home and cycling
7. Wed-Fri during the day at the HSLU T&A in Horw, in the evening in my flat; Sat-Sun in the flat, on the train and in the restaurant
8. Almost always.
9. During the day at school and at home from approx. 8:00 am to 9:00 pm
10. Day 1: at school, day 2,3: on the building site outside, day 4: sports outside, day 5: quiet day at home
11. University, home, and in between (public transport)
12. school, studio, home, nature, public transport
13. Work, school, driving
14. school (HSLU / private work / study)
15. Work
16. Wed - Fri at university, Saturday at home and Sunday on an excursion
17. When: Wednesday to Sunday, from 7-8am to 11pm; Where: At home, in the office, on the train, in the car
18. The Lido was mainly worn at university, as well as at home. This is because wearing it outside was usually made impossible by rain/snow
19. General housework, at home, working on the computer, leisure time
20. From Wednesday to Friday at university and Saturday and Sunday privately in all situations and occasions.
21. At school, while eating, at home and while studying
22. During work, on walks, with friends at home

23. At work and during all leisure activities
24. daily except for showering and sports
25. At work (home office), on the bike, at home in the morning and evening,
26. at home, in class (university), while hiking (Rigi with fog), restaurant, canteen, in the park, on walks
27. After getting "ready" for work or similar until the evening before getting "ready". Exceptions: professional appointments (external), sports, dates, in case of pain after wearing glasses for a long time, showering
28. As a spectacle wearer, from morning to evening, except when showering and sleeping
29. All day, studying, working on the PC

What activities did you carry out while you were wearing it?

1. working on the computer (school), mobile phone, and on the road for the club
2. working on the laptop, studying, working, watching Netflix, driving
3. driving, cycling, sitting at school, studying, working on the pc, watching tv,
4. contact study, self-study, cycling, travelling by train
5. Running the household, looking after the baby, walking, shopping, driving, working
6. lectures, working on projects, being at home a lot at weekends
7. computer work and working in the studio
8. lectures, self-study, skiing
9. laptop work, housework, shopping
10. Sitting at school, outdoors on the building site on the roof, cycling in the woods and in the fields, relaxing at home on the sofa in front of the TV
11. studying, eating, cooking, brushing teeth
12. learning, studying, reading, hacking, watching films
13. work, school, leisure, sport
14. learning, school
15. Working, walking the dog, family get-togethers
16. working on the laptop, watching videos on the mobile phone, cooking, handicrafts
17. working, watching films on the computer, driving, tidying up, cooking
18. studying, learning, cooking, walking, cycling
19. everyday tasks, often indoors because of the temperature
20. Shopping, working in the bar, bookkeeping, phone calls, working on student projects, group work, lectures, writing texts and emails, using mobile phones, watching Netflix & Youtube, walking, visiting grandmother, birthday party with friends, giving a talk at school, reading, train travel, working on the computer, making music with friends, eating in a restaurant, presentation from a company, discussions and one-to-one conversations, housework at home, spending time with the wife, eating, Zoom meetings and general meetings
21. school, studying, cycling, train travel and cooking
22. Working in front of the PC, walking, working in a ventilation duct, workshop work, cooking, meeting friends
23. office work, driving, making music
24. train travel, working on the computer
25. computer work, cooking, reading Ipad / mobile phone, cycling (as a means of transport)
26. Learning, walking, working on emails, watching films, cooking, watching lectures, baking, dancing, blow-drying hair, yoga
27. working, hiking, eating out, walking, watching TV, housework, sunbathing
28. commuting, school, eating etc. (no special activities)
29. studying, working on the PC, travelling by public transport, normal household chores, social media consumption

What were the reasons for not wearing the LiDo, apart from sleeping and showering?

1. I had a slight headache from time to time due to the pressure of the Lido and therefore took a break, otherwise I had a private appointment twice, it also rained from time to time
2. lecture at the university, restaurant visit
3. sports, family dinner
4. Saturday evening out
5. I always wore it
6. sports, Rain when cycling, in the evening it always started to pinch and I sometimes got a headache
7. swimming in the lake & sleeping during the day (Wednesday), make-up, sport (jogging), charging the Lido (Sunday afternoon)
8. When skiing, the bad weather and the poor fastening. When doing sports, the wobbling of the glasses and the risk of damage.
9. Sports activities, rain
10. rain when cycling, heavy rain when working on the roof
11. waiting for hair to dry, sports, going out
12. sometimes very heavy headache on the side where the Lido was worn
13. sports
14. cinema, family dinner, customer appointments
15. important business appointments
16. sports
17. fixing hair (hairstyle), charging, Conference
18. Rain + snow outside, which happened frequently these last few days
19. Business lunches, often short sleep intervals because I was ill
20. Cleaning glasses, lying awake in bed, transition from sleeping to showering, washing face
21. Presentation and charging the device
22. Sports, chilling, going out, glasses caused pain
23. Not used to glasses (headache when wearing). Glasses scratched
24. sports, presentations, modelling
25. sports (swimming, jogging), social gatherings and meetings
26. pain on the nose (rarely behind the right ear, i.e. on the side where I wore the Lido), for blow-drying hair/hair straightener, when applying make-up, face cream, sometimes when stretching, when running in the rain (on the Rigi), on the 4th day the Lido glowed red. On the fourth day, the Lido lit up red and I charged it for about 1.5 hours, during which I went for a walk and cooked
27. In case of pain (pressure points on the ear, nose and headache) sometimes a few minutes to an hour, date (several hours), professional customer appointment (1.5 h or similar)
28. Over time it became uncomfortable. As the device is "heavy", my glasses were a bit askew and it pressed on my ear
29. headache caused by the LiDo, sport, charging the device, hiking

On what occasion did you find it particularly difficult to wear your LiDo?

1. never, only when wearing it for longer periods of the day (I don't wear glasses, which was certainly a factor)
2. in public
3. in public, in town or on public transport
4. Never
5. none, I usually forget I'm wearing it
6. in the evening watching TV/ lying down
7. public places with lots of people

8. skiing
9. when shopping and trying on clothes it's a bit awkward
10. for personal reasons I never took off the LiDo. Only for the safety of the device. The only time I took it off was when I was changing so that it didn't get caught in my clothes.
11. going out (not worn), sports (partially worn)
12. presentations, public transport
13. when driving
14. in public
15. at important appointments it was rather heavy
16. sports, as it was too risky for me to avoid breaking it
17. at work, as it hurt my ear over time
18. outdoors in general and after longer periods of wearing it became heavier
19. When assembling a cupboard, my glasses often slipped off my nose
20. Never really, it was unpleasant to answer the same question many times about what the device was for
21. at school
22. sports, when chilling out (glasses hurt because LiDo is rather heavy)
23. when doing handwritten work. Glasses hung askew.
24. Headaches
25. in public
26. in the shop when people looked at me funny, when stretching (gravity), hiking (the Lido sometimes wobbles when you walk), in the evening because it was already very heavy
27. in the restaurant (uncomfortable), when walking/running (distorted vision, wobbling)
28. when running fast
29. travelling on public transport, situations in which I don't normally wear my glasses

What experiences (your own experience and possibly also the reactions of other people) did you have while wearing the device? Please briefly describe your observations!

1. generally very positive, many people thought it was a camera at first, but this was quickly clarified.
2. they looked and asked what it was and found it interesting. for others it also looks like a camera.
3. many people thought it was a camera. I was approached directly by everyone who saw it, nobody just accepted it without questioning it. Wearing them was not comfortable, after 4-6 hours the glasses were very tight on one side.
4. many looks. I was asked a lot of questions and a lot of interest. Of course, I then explained what the light collector was all about.
5. I was only approached by people who know me. They were all relieved that it wasn't a camera. But a camera was always the first thought. Other people just looked at me sceptically but didn't say anything. I myself usually didn't even think that I was wearing it.
6. people often thought that I was filming them, many people just looked confused or asked what it was
7. no big ones, people I knew asked what it was exactly, no reaction from strangers.
8. About 50/50 some people said that they could sign up directly and the other half said it looked stupid, they would never wear it.
9. When walking through the city, some people looked at you longer than usual.
10. Surprisingly unobtrusive for someone who doesn't usually wear glasses. The device on the side is quite noticeable, which is often pointed out. While wearing the device, I have become a little more aware of the light I expose myself to throughout the day.
11. pressure of the glasses on the nose, curiosity of others about the LiDo, questions like "You wear glasses?"

12. a lot of "gawking" and funny looks (especially on public transport and especially from older people). Some people were afraid that they were being filmed.
13. asked about the purpose of the device and its function.
14. everyone looked at what was on the glasses
15. lots of people asked me what kind of device it was.
16. almost every person I spoke to asked me what the LiDo was. It was easier than I thought to get used to the device.
17. every employee noticed it and asked what it was. They suspected that I was recording something. After clarification, everything was clear and some people on the train looked at me strangely.
18. there was constant curiosity from other people but the Lido could usually be explained in 2-3 sentences.
19. people asked what kind of device it was.
20. a lot of people asked what the device was for (friends, fellow students, family members, acquaintances, strangers), a few strange looks from strangers, when it rained with a cap you had to make sure that the transmitter was not covered, the device is relatively heavy and caused an imbalance in my otherwise very stable glasses, the glasses were slightly bent and therefore had to be readjusted, some of the flashing was very bright and irritating. Some of the flashing was very bright and irritating in dark situations, I had to adapt my behaviour (e.g. no participation in snowball fights), people often thought it was a camera, I thought about when wearing it would lead to fewer complications beforehand
21. You are often looked at by other passers-by and they often ask what this device is all about. The glasses get a little heavier when you wear them, which makes wearing them a little more difficult as the glasses always slide down.
22. People avoid eye contact (probably think it's a camera), friends smiled at how it looks
23. No negative comments. Lots of interest.
24. many reacted with irritation and thought the device was a camera
25. irritation and increased attention. For example, I passed a group of traffic police officers who were having a meeting at the side of the road. I wanted to pass them and cross the road, but as the traffic lights were red I changed direction. The policemen stopped talking and turned round to look at me, they probably thought I was filming them...
26. I was watched more, strangers on the train/in the shop behaved differently (looked at me more, sometimes asked if they were on TV or directly "is that a camera"? and looked away.) I myself therefore perceived it mainly as an "increase in attention", sometimes I didn't care, sometimes I was a bit uncomfortable and I would have liked to have taken it off. I also quickly noticed that the glasses hung at a slight angle due to the weight. They also wobbled when I walked, which was rather annoying. Other reactions from others were: "How long are you wearing that now?", "Don't you want to put a counterweight on the other side?", "Isn't that really heavy?", "Do you really think there's no camera or microphone on it?", "So what exactly is it measuring now?")
27. received and accepted with humour/interest by those around me, no "funny" looks as I might have expected. Not approached by any strangers.
28. Basically it was okay. The device is a bit conspicuous and people stared at me, especially on the train (perhaps because it looks more like a camera). Wearing it was also okay, but after a while it became uncomfortable because the weight of the glasses made them slanted and so it became uncomfortable on the ear. Apart from that, it wasn't too annoying. When I told people why, they were delighted.
29. It's hardly noticeable when you're sitting down. When walking it is very annoying as it makes the whole glasses bounce/swing. The LiDo appears in the field of vision and you also notice the spectacle frame. The device attracts a lot of attention, strangers watch you and even stare at you. The environment is irritated/distracted by the LiDo. People (on campus) have laughed out loud at me because it looks pretty stupid. Strangers on public transport think it's a camera and there were situations where others changed seats and didn't want to sit opposite me.
