# Supplementary material for: Wearable Light Loggers in Field Conditions: Corneal Light Characteristics, User Compliance, and Acceptance
Source: Clocks Sleep. 2024 Oct 25;6(4):619–34. doi: 10.3390/clockssleep6040042 (PMC11586969; doi:10.3390/clockssleep6040042)
Supplement: Supplementary file 1 [file clockssleep-06-00042-s001.zip › SupplementS2_Chronotype_Analysis.html]

Supplement S2


## Table of contents

- 1 Preface
- 2 Summary
- 3 Setup
- 4 Import
- 5 Statistical analysis
  - 5.1 Constructing the different model types
  - 5.2 Model comparison

# Supplement S2

 Code

- Show All Code
- Hide All Code
- ---
- View Source

Author

Johannes Zauner

## 1 Preface

This analysis is part of of the publication “Wearable light loggers in field conditions: Corneal light characteristics, user compliance and acceptance” by Oliver Stefani, Reto Marek, Jürg Schwarz, Sina Plate, Johannes Zauner, and Björn Schrader. The main goal of this analysis is to analyze whether there are differences in the daily light exposure patterns between different chronotypes in the study.

The analysis is performed with Generalized Additive Mixed models. Five model types are constructed and compared according to *Pedersen, E. J., Miller, D. L., Simpson, G. L., & Ross, N. (2019). Hierarchical generalized additive models in ecology: an introduction with mgcv. PeerJ, 7, e6876. https://doi.org/10.7717/peerj.6876*. The main goal of this comparison is to determine whether the light exposure follows

- an overall pattern with no differences between chronotypes (model G)
- an overall pattern with differences between chronotypes, but identical smoothing parameters (model GS)
- an overall pattern with differences between chronotypes, and differing smoothing parameters (model GI)
- no overall pattern, but differences between chronotypes with identical smoothing parameters (model S)
- no overall pattern, but differences between chronotypes with differing smoothing parameters (model I)

Data were cleaned and pre-processed prior to analysis by O.S.

## 2 Summary

Based on AIC a global smooth is suggested. While the lowest AIC scores go to models that factor in Chronotype, the difference between chronotypes is not significant. While more complex models could factor in covariates like weekend/weekday, overall the differences in light exposure do not differ between the chronotypes.

## 3 Setup

Code

```
library(LightLogR)
library(tidyverse)
```

```
── Attaching core tidyverse packages ──────────────────────── tidyverse 2.0.0 ──
✔ dplyr     1.1.4     ✔ readr     2.1.5
✔ forcats   1.0.0     ✔ stringr   1.5.1
✔ ggplot2   3.5.1     ✔ tibble    3.2.1
✔ lubridate 1.9.3     ✔ tidyr     1.3.1
✔ purrr     1.0.2     
── Conflicts ────────────────────────────────────────── tidyverse_conflicts() ──
✖ dplyr::filter() masks stats::filter()
✖ dplyr::lag()    masks stats::lag()
ℹ Use the conflicted package (<http://conflicted.r-lib.org/>) to force all conflicts to become errors
```

Code

```
library(mgcv)
```

```
Loading required package: nlme

Attaching package: 'nlme'

The following object is masked from 'package:dplyr':

    collapse

This is mgcv 1.9-1. For overview type 'help("mgcv-package")'.
```

Code

```
library(itsadug)
```

```
Loading required package: plotfunctions

Attaching package: 'plotfunctions'

The following object is masked from 'package:ggplot2':

    alpha

Loaded package itsadug 2.4 (see 'help("itsadug")' ).
```

## 4 Import

Code

```
load("rounded_mEDI_30min_chrono.RData")

#prepare the dataframe
df <- df %>% mutate(across(c(Id, chronotype), factor),
                    Date = date(Timestamp_rounded),
                    Time = hms::as_hms(Timestamp_rounded))

#remove (5x) duplicate rows
df <- df %>% distinct()

#make Date a factor
df <- df %>% mutate(Date = factor(Date))
#make a time variable as decimal-hours
df <- df %>% mutate(Time_h = as.numeric(Time) / 3600)

#create an interaction for Id and Date
df <- df %>% mutate(Id_Date = interaction(Id, Date))

df$chronotype %>% unique()
```

```
[1] moderate evening moderate morning intermediate    
Levels: intermediate moderate evening moderate morning
```

Code

```
#make chronotype an ordered factor
df <- df %>% mutate(chronotype = factor(chronotype, ordered = TRUE, 
                                        levels = c("moderate evening", "intermediate", "moderate morning")))
```

## 5 Statistical analysis

Setting up some global variables for the analysis

Code

```
#setting the ends for the cyclic smooth
knots_day <- list(Time_h = c(0, 24))
```

### 5.1 Constructing the different model types

#### 5.1.1 Model 1: Global smooth (G)

Code

```
formula_G <- log10(value) ~ s(Time_h, bs = "cc", k=10, m=2) + s(Id_Date, bs = "re")

chrono_modG <- gam(formula_G, data = df,
                    method = "REML", knots = knots_day)

chrono_modG_sum <- summary(chrono_modG)

chrono_modG_sum
```

```
Family: gaussian 
Link function: identity 

Formula:
log10(value) ~ s(Time_h, bs = "cc", k = 10, m = 2) + s(Id_Date, 
    bs = "re")

Parametric coefficients:
            Estimate Std. Error t value Pr(>|t|)    
(Intercept)  1.90025    0.03024   62.84   <2e-16 ***
---
Signif. codes:  0 '***' 0.001 '**' 0.01 '*' 0.05 '.' 0.1 ' ' 1

Approximate significance of smooth terms:
               edf Ref.df       F p-value    
s(Time_h)    7.162      8 438.517  <2e-16 ***
s(Id_Date) 101.955    119   5.612  <2e-16 ***
---
Signif. codes:  0 '***' 0.001 '**' 0.01 '*' 0.05 '.' 0.1 ' ' 1

R-sq.(adj) =  0.553   Deviance explained = 57.6%
-REML = 1585.6  Scale est. = 0.22614   n = 2138
```

Code

```
gam.check(chrono_modG, rep = 500)
```

```
Method: REML   Optimizer: outer newton
full convergence after 7 iterations.
Gradient range [-0.0003448715,8.098026e-05]
(score 1585.608 & scale 0.2261359).
Hessian positive definite, eigenvalue range [2.166476,1071.03].
Model rank =  129 / 129 

Basis dimension (k) checking results. Low p-value (k-index<1) may
indicate that k is too low, especially if edf is close to k'.

               k'    edf k-index p-value
s(Time_h)    8.00   7.16    0.98    0.18
s(Id_Date) 120.00 101.96      NA      NA
```

Code

```
#Model overview
plot(chrono_modG, shade = TRUE, residuals = TRUE, cex = 1, all.terms = TRUE)
```

#### 5.1.2 Model 2: Global smooth + group-level smoother with identical smoothing parameter (GS)

Code

```
formula_GS <- log10(value) ~ s(Time_h, bs = "cc") + s(Time_h,chronotype, k=10, bs = "fs") + s(Id_Date, bs = "re") 

chrono_modGS <- gam(formula_GS, data = df,
                    method = "REML", knots = knots_day)
```

```
Warning in gam.side(sm, X, tol = .Machine$double.eps^0.5): model has repeated
1-d smooths of same variable.
```

Code

```
chrono_modGS_sum <- 
summary(chrono_modGS)
chrono_modGS_sum
```

```
Family: gaussian 
Link function: identity 

Formula:
log10(value) ~ s(Time_h, bs = "cc") + s(Time_h, chronotype, k = 10, 
    bs = "fs") + s(Id_Date, bs = "re")

Parametric coefficients:
            Estimate Std. Error t value Pr(>|t|)    
(Intercept)  1.91027    0.04359   43.83   <2e-16 ***
---
Signif. codes:  0 '***' 0.001 '**' 0.01 '*' 0.05 '.' 0.1 ' ' 1

Approximate significance of smooth terms:
                         edf Ref.df       F p-value    
s(Time_h)              7.022      8 275.728  <2e-16 ***
s(Time_h,chronotype)   2.990     29   1.635   0.213    
s(Id_Date)           101.315    119   5.605  <2e-16 ***
---
Signif. codes:  0 '***' 0.001 '**' 0.01 '*' 0.05 '.' 0.1 ' ' 1

R-sq.(adj) =  0.556   Deviance explained = 57.9%
-REML = 1580.2  Scale est. = 0.2244    n = 2138
```

Code

```
gam.check(chrono_modGS, rep = 500)
```

```
Method: REML   Optimizer: outer newton
full convergence after 7 iterations.
Gradient range [-0.0007156785,0.0001765162]
(score 1580.168 & scale 0.224402).
Hessian positive definite, eigenvalue range [0.0001193878,1070.999].
Model rank =  159 / 159 

Basis dimension (k) checking results. Low p-value (k-index<1) may
indicate that k is too low, especially if edf is close to k'.

                         k'    edf k-index p-value
s(Time_h)              8.00   7.02    0.99    0.29
s(Time_h,chronotype)  30.00   2.99    0.99    0.19
s(Id_Date)           120.00 101.32      NA      NA
```

Code

```
#Model overview
plot(chrono_modGS, shade = TRUE, residuals = TRUE, cex = 1, all.terms = TRUE)
```

Code

```
plot_diff(chrono_modGS,
          view = "Time_h", rm.ranef = "s(Id_Date)",
          comp = list(chronotype = c("moderate morning", "moderate evening")),
          sim.ci = TRUE)
```

```
Warning in mgcv::predict.gam(model, newd1, type = "lpmatrix"): factor levels
UX07.2023-10-29 not in original fit
```

```
Warning in mgcv::predict.gam(model, newd2, type = "lpmatrix"): factor levels
UX07.2023-10-29 not in original fit
```

```
Summary:
    * Time_h : numeric predictor; with 200 values ranging from 0.000000 to 23.500000. 
    * Id_Date : factor; set to the value(s): UX07.2023-10-29. (Might be canceled as random effect, check below.) 
    * NOTE : The following random effects columns are canceled: s(Time_h),s(Time_h,chronotype),s(Id_Date)
 
    * Simultaneous 95%-CI used : 
        Critical value: 2.319
        Proportion posterior simulations in pointwise CI: 1 (10000 samples)
        Proportion posterior simulations in simultaneous CI: 1 (10000 samples)
```

```
Difference is not significant.
```

#### 5.1.3 Model 3: Global smooth + group-level smoother with differing smoothing parameter (GI)

Code

```
formula_GI <- log10(value) ~ chronotype + s(Time_h, bs = "cc") + s(Time_h, by = chronotype, bs = "cc") + s(Id_Date, bs = "re") 

chrono_modGI <- gam(formula_GI, data = df,
                    method = "REML", knots = knots_day)

chrono_modGI_sum <- 
summary(chrono_modGI)

chrono_modGI_sum
```

```
Family: gaussian 
Link function: identity 

Formula:
log10(value) ~ chronotype + s(Time_h, bs = "cc") + s(Time_h, 
    by = chronotype, bs = "cc") + s(Id_Date, bs = "re")

Parametric coefficients:
             Estimate Std. Error t value Pr(>|t|)    
(Intercept)   1.93316    0.03572  54.112   <2e-16 ***
chronotype.L -0.11789    0.06984  -1.688   0.0916 .  
chronotype.Q  0.07022    0.05288   1.328   0.1844    
---
Signif. codes:  0 '***' 0.001 '**' 0.01 '*' 0.05 '.' 0.1 ' ' 1

Approximate significance of smooth terms:
                                           edf Ref.df       F  p-value    
s(Time_h)                            7.077e+00      8 302.088  < 2e-16 ***
s(Time_h):chronotypeintermediate     8.959e-04      8   0.000 0.918483    
s(Time_h):chronotypemoderate morning 2.330e+00      8   2.876 0.000397 ***
s(Id_Date)                           1.003e+02    117   5.664  < 2e-16 ***
---
Signif. codes:  0 '***' 0.001 '**' 0.01 '*' 0.05 '.' 0.1 ' ' 1

R-sq.(adj) =  0.556   Deviance explained = 57.9%
-REML = 1583.2  Scale est. = 0.22457   n = 2138
```

Code

```
gam.check(chrono_modGI, rep = 500)
```

```
Method: REML   Optimizer: outer newton
full convergence after 10 iterations.
Gradient range [-0.0003859047,6.215361e-05]
(score 1583.167 & scale 0.2245708).
Hessian positive definite, eigenvalue range [0.0003857564,1069.95].
Model rank =  147 / 147 

Basis dimension (k) checking results. Low p-value (k-index<1) may
indicate that k is too low, especially if edf is close to k'.

                                           k'      edf k-index p-value
s(Time_h)                            8.00e+00 7.08e+00    0.99    0.23
s(Time_h):chronotypeintermediate     8.00e+00 8.96e-04    0.99    0.28
s(Time_h):chronotypemoderate morning 8.00e+00 2.33e+00    0.99    0.24
s(Id_Date)                           1.20e+02 1.00e+02      NA      NA
```

Code

```
#Model overview
plot(chrono_modGI, shade = TRUE, residuals = TRUE, cex = 1, all.terms = TRUE)
```

Code

```
plot_diff(chrono_modGI,
          view = "Time_h", rm.ranef = "s(Id_Date)",
          comp = list(chronotype = c("moderate morning", "moderate evening")),
          sim.ci = TRUE)
```

```
Warning in mgcv::predict.gam(model, newd1, type = "lpmatrix"): factor levels
UX07.2023-10-29 not in original fit
```

```
Warning in mgcv::predict.gam(model, newd2, type = "lpmatrix"): factor levels
UX07.2023-10-29 not in original fit
```

```
Summary:
    * Time_h : numeric predictor; with 200 values ranging from 0.000000 to 23.500000. 
    * Id_Date : factor; set to the value(s): UX07.2023-10-29. (Might be canceled as random effect, check below.) 
    * NOTE : The following random effects columns are canceled: s(Time_h),s(Time_h):chronotypeintermediate,s(Time_h):chronotypemoderate morning,s(Id_Date)
 
    * Simultaneous 95%-CI used : 
        Critical value: 2.559
        Proportion posterior simulations in pointwise CI: 1 (10000 samples)
        Proportion posterior simulations in simultaneous CI: 1 (10000 samples)
```

```
Difference is not significant.
```

#### 5.1.4 Model 4: No Global smooth + group-level smoother with identical smoothing parameter (S)

Code

```
formula_S <- log10(value) ~ s(Time_h, chronotype, bs = "fs") + s(Id_Date, bs = "re") 

chrono_modS <- gam(formula_S, data = df %>% drop_na(),
                    method = "REML", knots = knots_day)

chrono_modS_sum <- 
summary(chrono_modS)

chrono_modS_sum
```

```
Family: gaussian 
Link function: identity 

Formula:
log10(value) ~ s(Time_h, chronotype, bs = "fs") + s(Id_Date, 
    bs = "re")

Parametric coefficients:
            Estimate Std. Error t value Pr(>|t|)    
(Intercept)  1.83146    0.03047   60.12   <2e-16 ***
---
Signif. codes:  0 '***' 0.001 '**' 0.01 '*' 0.05 '.' 0.1 ' ' 1

Approximate significance of smooth terms:
                        edf Ref.df       F p-value    
s(Time_h,chronotype)  19.14     29 122.849  <2e-16 ***
s(Id_Date)           101.80    119   5.558  <2e-16 ***
---
Signif. codes:  0 '***' 0.001 '**' 0.01 '*' 0.05 '.' 0.1 ' ' 1

R-sq.(adj) =  0.555   Deviance explained =   58%
-REML = 1598.6  Scale est. = 0.2251    n = 2138
```

Code

```
gam.check(chrono_modS, rep = 500)
```

```
Method: REML   Optimizer: outer newton
full convergence after 8 iterations.
Gradient range [-0.001430439,0.002355149]
(score 1598.613 & scale 0.2251006).
Hessian positive definite, eigenvalue range [0.0001185047,1071.071].
Model rank =  151 / 151 

Basis dimension (k) checking results. Low p-value (k-index<1) may
indicate that k is too low, especially if edf is close to k'.

                        k'   edf k-index p-value
s(Time_h,chronotype)  30.0  19.1    0.99    0.28
s(Id_Date)           120.0 101.8      NA      NA
```

Code

```
#Model overview
plot(chrono_modS, shade = TRUE, residuals = TRUE, cex = 1, all.terms = TRUE)
```

Code

```
plot_diff(chrono_modS,
          view = "Time_h", rm.ranef = "s(Id_Date)",
          comp = list(chronotype = c("moderate morning", "moderate evening")),
          sim.ci = TRUE)
```

```
Summary:
    * Time_h : numeric predictor; with 200 values ranging from 0.000000 to 23.500000. 
    * Id_Date : factor; set to the value(s): UX21.2023-11-24. (Might be canceled as random effect, check below.) 
    * NOTE : The following random effects columns are canceled: s(Time_h,chronotype),s(Id_Date)
 
    * Simultaneous 95%-CI used : 
        Critical value: 2.984
        Proportion posterior simulations in pointwise CI: 1 (10000 samples)
        Proportion posterior simulations in simultaneous CI: 1 (10000 samples)
```

```
Difference is not significant.
```

#### 5.1.5 Model 5: No Global smooth + group-level smoother with different smoothing parameter (I)

Code

```
formula_I <- log10(value) ~ chronotype + s(Time_h, by = chronotype, bs = "cc") + s(Id_Date, bs = "re") 

chrono_modI <- gam(formula_I, data = df,
                    method = "REML", knots = knots_day)

chrono_modI_sum <- 
summary(chrono_modI)

chrono_modI_sum
```

```
Family: gaussian 
Link function: identity 

Formula:
log10(value) ~ chronotype + s(Time_h, by = chronotype, bs = "cc") + 
    s(Id_Date, bs = "re")

Parametric coefficients:
             Estimate Std. Error t value Pr(>|t|)    
(Intercept)   1.91284    0.03885  49.240   <2e-16 ***
chronotype.L -0.07245    0.07580  -0.956    0.339    
chronotype.Q  0.04602    0.05752   0.800    0.424    
---
Signif. codes:  0 '***' 0.001 '**' 0.01 '*' 0.05 '.' 0.1 ' ' 1

Approximate significance of smooth terms:
                                         edf Ref.df       F p-value    
s(Time_h):chronotypeintermediate       6.329      8 229.081  <2e-16 ***
s(Time_h):chronotypemoderate morning   4.925      8 131.727  <2e-16 ***
s(Id_Date)                           101.665    117   6.131  <2e-16 ***
---
Signif. codes:  0 '***' 0.001 '**' 0.01 '*' 0.05 '.' 0.1 ' ' 1

R-sq.(adj) =   0.52   Deviance explained = 54.6%
-REML = 1673.9  Scale est. = 0.24279   n = 2138
```

Code

```
gam.check(chrono_modI, rep = 500)
```

```
Method: REML   Optimizer: outer newton
full convergence after 7 iterations.
Gradient range [-4.316696e-05,1.886303e-05]
(score 1673.86 & scale 0.24279).
Hessian positive definite, eigenvalue range [1.992211,1070.023].
Model rank =  139 / 139 

Basis dimension (k) checking results. Low p-value (k-index<1) may
indicate that k is too low, especially if edf is close to k'.

                                         k'    edf k-index p-value   
s(Time_h):chronotypeintermediate       8.00   6.33    0.94   0.005 **
s(Time_h):chronotypemoderate morning   8.00   4.93    0.94   0.010 **
s(Id_Date)                           120.00 101.66      NA      NA   
---
Signif. codes:  0 '***' 0.001 '**' 0.01 '*' 0.05 '.' 0.1 ' ' 1
```

Code

```
#Model overview
plot(chrono_modI, shade = TRUE, residuals = TRUE, cex = 1, all.terms = TRUE)
```

Code

```
plot_diff(chrono_modI,
          view = "Time_h", rm.ranef = "s(Id_Date)",
          comp = list(chronotype = c("moderate morning", "moderate evening")),
          sim.ci = TRUE)
```

```
Warning in mgcv::predict.gam(model, newd1, type = "lpmatrix"): factor levels
UX07.2023-10-29 not in original fit
```

```
Warning in mgcv::predict.gam(model, newd2, type = "lpmatrix"): factor levels
UX07.2023-10-29 not in original fit
```

```
Summary:
    * Time_h : numeric predictor; with 200 values ranging from 0.000000 to 23.500000. 
    * Id_Date : factor; set to the value(s): UX07.2023-10-29. (Might be canceled as random effect, check below.) 
    * NOTE : The following random effects columns are canceled: s(Time_h):chronotypeintermediate,s(Time_h):chronotypemoderate morning,s(Id_Date)
 
    * Simultaneous 95%-CI used : 
        Critical value: 2.685
        Proportion posterior simulations in pointwise CI: 1 (10000 samples)
        Proportion posterior simulations in simultaneous CI: 1 (10000 samples)
```

```
Time_h window(s) of significant difference(s):
    0.000000 - 3.660804
    10.155779 - 13.108040
    17.477387 - 23.500000
```

### 5.2 Model comparison

Code

```
AIC(chrono_modG, chrono_modGS, chrono_modGI, chrono_modS, chrono_modI)
```

```
                   df      AIC
chrono_modG  112.0558 3000.047
chrono_modGS 115.9850 2989.117
chrono_modGI 115.8150 2990.000
chrono_modS  124.0941 3001.812
chrono_modI  118.0087 3157.755
```

##### Source Code

```
---
title: "Supplement S2"
author: "Johannes Zauner"
format: 
  html:
    self-contained: true
    toc: true
    number-sections: true
    code-overflow: wrap
    code-link: true
    code-tools: true
    code-fold: TRUE
---

## Preface

This analysis is part of of the publication "Wearable light loggers in field conditions: Corneal light characteristics, user compliance and acceptance" by Oliver Stefani, Reto Marek, Jürg Schwarz, Sina Plate, Johannes Zauner, and Björn Schrader. The main goal of this analysis is to analyze whether there are differences in the daily light exposure patterns between different chronotypes in the study.

The analysis is performed with Generalized Additive Mixed models. Five model types are constructed and compared according to *Pedersen, E. J., Miller, D. L., Simpson, G. L., & Ross, N. (2019). Hierarchical generalized additive models in ecology: an introduction with mgcv. PeerJ, 7, e6876. https://doi.org/10.7717/peerj.6876*. The main goal of this comparison is to determine whether the light exposure follows 

-   an overall pattern with no differences between chronotypes (model G)
-   an overall pattern with differences between chronotypes, but identical smoothing parameters (model GS)
-   an overall pattern with differences between chronotypes, and differing smoothing parameters (model GI)
-   no overall pattern, but differences between chronotypes with identical smoothing parameters (model S)
-   no overall pattern, but differences between chronotypes with differing smoothing parameters (model I)

Data were cleaned and pre-processed prior to analysis by O.S.

## Summary

Based on AIC a global smooth is suggested. While the lowest AIC scores go to models that factor in Chronotype, the difference between chronotypes is not significant. While more complex models could factor in covariates like weekend/weekday, overall the differences in light exposure do not differ between the chronotypes.

## Setup

```{r}
library(LightLogR)
library(tidyverse)
library(mgcv)
library(itsadug)
```
## Import

```{r}
load("rounded_mEDI_30min_chrono.RData")

#prepare the dataframe
df <- df %>% mutate(across(c(Id, chronotype), factor),
                    Date = date(Timestamp_rounded),
                    Time = hms::as_hms(Timestamp_rounded))

#remove (5x) duplicate rows
df <- df %>% distinct()

#make Date a factor
df <- df %>% mutate(Date = factor(Date))
#make a time variable as decimal-hours
df <- df %>% mutate(Time_h = as.numeric(Time) / 3600)

#create an interaction for Id and Date
df <- df %>% mutate(Id_Date = interaction(Id, Date))

df$chronotype %>% unique()

#make chronotype an ordered factor
df <- df %>% mutate(chronotype = factor(chronotype, ordered = TRUE, 
                                        levels = c("moderate evening", "intermediate", "moderate morning")))

```

## Statistical analysis

Setting up some global variables for the analysis

```{r}
#setting the ends for the cyclic smooth
knots_day <- list(Time_h = c(0, 24))
```

### Constructing the different model types

#### Model 1: Global smooth (G)

```{r}
formula_G <- log10(value) ~ s(Time_h, bs = "cc", k=10, m=2) + s(Id_Date, bs = "re")

chrono_modG <- gam(formula_G, data = df,
                    method = "REML", knots = knots_day)

chrono_modG_sum <- summary(chrono_modG)

chrono_modG_sum

gam.check(chrono_modG, rep = 500)

#Model overview
plot(chrono_modG, shade = TRUE, residuals = TRUE, cex = 1, all.terms = TRUE)
```

#### Model 2: Global smooth + group-level smoother with identical smoothing parameter (GS)

```{r}
formula_GS <- log10(value) ~ s(Time_h, bs = "cc") + s(Time_h,chronotype, k=10, bs = "fs") + s(Id_Date, bs = "re") 

chrono_modGS <- gam(formula_GS, data = df,
                    method = "REML", knots = knots_day)

chrono_modGS_sum <- 
summary(chrono_modGS)
chrono_modGS_sum

gam.check(chrono_modGS, rep = 500)

#Model overview
plot(chrono_modGS, shade = TRUE, residuals = TRUE, cex = 1, all.terms = TRUE)
plot_diff(chrono_modGS,
          view = "Time_h", rm.ranef = "s(Id_Date)",
          comp = list(chronotype = c("moderate morning", "moderate evening")),
          sim.ci = TRUE)


```

#### Model 3: Global smooth + group-level smoother with differing smoothing parameter (GI)

```{r}
formula_GI <- log10(value) ~ chronotype + s(Time_h, bs = "cc") + s(Time_h, by = chronotype, bs = "cc") + s(Id_Date, bs = "re") 

chrono_modGI <- gam(formula_GI, data = df,
                    method = "REML", knots = knots_day)

chrono_modGI_sum <- 
summary(chrono_modGI)

chrono_modGI_sum

gam.check(chrono_modGI, rep = 500)

#Model overview
plot(chrono_modGI, shade = TRUE, residuals = TRUE, cex = 1, all.terms = TRUE)

plot_diff(chrono_modGI,
          view = "Time_h", rm.ranef = "s(Id_Date)",
          comp = list(chronotype = c("moderate morning", "moderate evening")),
          sim.ci = TRUE)


```


#### Model 4: No Global smooth + group-level smoother with identical smoothing parameter (S)

```{r}
formula_S <- log10(value) ~ s(Time_h, chronotype, bs = "fs") + s(Id_Date, bs = "re") 

chrono_modS <- gam(formula_S, data = df %>% drop_na(),
                    method = "REML", knots = knots_day)

chrono_modS_sum <- 
summary(chrono_modS)

chrono_modS_sum

gam.check(chrono_modS, rep = 500)

#Model overview
plot(chrono_modS, shade = TRUE, residuals = TRUE, cex = 1, all.terms = TRUE)

plot_diff(chrono_modS,
          view = "Time_h", rm.ranef = "s(Id_Date)",
          comp = list(chronotype = c("moderate morning", "moderate evening")),
          sim.ci = TRUE)

```


#### Model 5: No Global smooth + group-level smoother with different smoothing parameter (I)

```{r}
formula_I <- log10(value) ~ chronotype + s(Time_h, by = chronotype, bs = "cc") + s(Id_Date, bs = "re") 

chrono_modI <- gam(formula_I, data = df,
                    method = "REML", knots = knots_day)

chrono_modI_sum <- 
summary(chrono_modI)

chrono_modI_sum

gam.check(chrono_modI, rep = 500)

#Model overview
plot(chrono_modI, shade = TRUE, residuals = TRUE, cex = 1, all.terms = TRUE)

plot_diff(chrono_modI,
          view = "Time_h", rm.ranef = "s(Id_Date)",
          comp = list(chronotype = c("moderate morning", "moderate evening")),
          sim.ci = TRUE)

```


### Model comparison

```{r}
AIC(chrono_modG, chrono_modGS, chrono_modGI, chrono_modS, chrono_modI)
```
```
